# Book Notices

**Published:** 1889-10

**Authors:** 


ELECTRICAL DISTRIBUTION OF LIGHT, heat and power, with partial list of deaths from lightning circuits, by Harold P. B. Brown, electrical engineer, 45 Wall St., New York, and an address by John Murray Mitchell, counsellor at law, or Legislative control of dangerous electrical currents, New York. J. W. Pratt & Son, Pub., 79 Fulton St.

KILNER'S PHYSICIAN'S POCKET DAY BOOK, JOURNAL AND LEDGER.—This is certainly a unique and excellent day book and ledger for physicians. It is 4 inches wide by 7¼ long, and though sufficintly compact to be carried in a side coat pocket, it is large enough for the rnnning accounts of 400 persons. When the practitioner has once acquainted himself with the short hand method of making the entries, he will be always, with this book in his pocket, ready to show the exact amount of the account of any of his patrons. The one entry which he makes completes the whole work for the day book, journal and ledger. Price $2.00. Published by Dr. S. Kilner, South Bend, Ind.

PHYSICIAN'S LEISURE LIBRARY.--The following copies received:

On Disordered Digestion and Dyspepsia, by Frank Woodbury, A. M., M. D., Fellow of the College of Physicians, Honorary Professor of Clinical Medicine in the Medico-Chirurgical College of Phila., Physician to the Medico-Chirurgical Hospital etc. Geo. S. Davis, Pub., Detroit, Mich. Contains much useful and practical information on the subjects treated.

SYPHILIS OF THE NERVOUS SYSTEM, by H. H. C. Wood, M D., LL. D. Geo. Davis, Pub., Detroit, Mich.

The subject ably treated under the general heads of, Etiology; the brain and its membranes; Spinal syphilis; the peripheral nerves.

The work is largely the outcome of the author's personal experience. Deductions drawn from five thousand cases of nervous diseases in the University Hospital and Dispensary

ON THE TREATMENT OF THE MORPHINE HABIT, by Dr. Albrecht Erlenmeyer, Translated from the German. Pub. by Geo. S. Davis, Detroit, Mich.

This book is drawn from the great work by Dr. Albrecht Erlenmeyer on the Morphine habit. Publshed first in 1887.

It deals specially with the treatment, and is full of important instruction on this interesting subject. It treats of *Methods of withdrawal; where* to treat and the *means necessary to success. Symptoms developed by abstinence. The cocaine Treatment. Prevention of relapse*, etc.

TRAVELERS R. R. GUIDE, Sept. 1889. Knickerbocker Guide Co., 46 Bond St., New York. Large, full and complete on various routes, hotels, public resorts, etc., etc.

CLINICAL STUDY ON ALOPECEA AREATA, by K. Duncan Buckley, A. M., M. D., New York, Trow's Printing and Bookbinding Co. A well written and interesting pamphlet.

AMERICAN PUBLIC HEALTH ASSOCIATION, Brooklyn, 1889.—The Seventeenth annual meeting of this Association will be held in the Hall of the Brooklyn Institute, Washington and Concord Sts., October 22, 23, 24, 25.

Addresses of welcome will be delivered by Hon. Alfred C. Chapin, Mayor on behalf of the City, and by Alexander Hutchins, M. D., on behalf of the medical profession.

Many interesting topics have been selected for consideration at the meeting. Exhibition of sanitary goods and appliances in another large hall close by. Applicants for space should address

A. N. BELL, M. D., Chairman,
113 A. Second Place, Brooklyn, N. Y.

THE SOUTHERN SURGICAL AND GYNECOLOGICAL ASSOCIATION will be held in Nashville, Tenn., Nov. 12th, 13th and 14th, 1889.

President, Hunter McGuire, M. D., LL. D., Richmond, Virginia.

Chairman of the committee of arrangements, Duncan Eve, M. D , Nashville, Tennessee.

Numerous interesting papers to be read.

Members of the medical profession are cordially invited to attend.

THE OPERATIONS OF SURGERY.—A systematic handbook for practitioners, students and hospital surgeons, by W. H. A Jacobson, F. R. C. S., Assistant surgeon, Guy's Hospital; Teacher of operative surgery and joint teacher of practical surgery in the Medical School; Surgeon to the Royal Hospital for Children and Women, with one hundred and ninety-nine illustrations. Philadelphia. P. Blakiston, Son & Co., 1012 Walnut St.

We find this an able and very practical work, full and comyrehensive upon all important operations in surgery. It contains more than one thousand papers, and the illustrations are numerous and excellent.

The various operative procedures are considered under six principal parts as follows:

1. Operations on the upper extremity.
2. Operations on the head and neck.
3. Operations on the thorax.
4. Operations on the abdomen.
5. Operations on the lower extremity.
6. Operations on the urethral canal.

We recommend this work as eminently practical and up with the latest advances in operative surgery.

